# Spying on the boron–boron triple bond using spin–spin coupling measured from ^11^B solid-state NMR spectroscopy†
†Electronic supplementary information (ESI) available: Experimental and computational details. See DOI: 10.1039/c5sc00644a



**DOI:** 10.1039/c5sc00644a

**Published:** 2015-04-01

**Authors:** Frédéric A. Perras, William C. Ewing, Theresa Dellermann, Julian Böhnke, Stefan Ullrich, Thomas Schäfer, Holger Braunschweig, David L. Bryce

**Affiliations:** a Department of Chemistry and CCRI , University of Ottawa , 10 Marie Curie Pvt. D'Iorio Hall , Ottawa , Ontario K1N6N5 , Canada . Email: dbryce@uottawa.ca; b Institut für Anorganische Chemie , Julius-Maximilians-Universität Würzburg , Am Hubland , 97074 , Germany . Email: h.braunschweig@uni-wuerzburg.de

## Abstract

There is currently tremendous interest in the previously documented example of a stable species exhibiting a boron–boron triple bond (*Science*, 2012, **336**, 1420). Notably, it has recently been stated using arguments based on force constants that this diboryne may not, in reality, feature a boron–boron triple bond. Here, we use advanced solid-state NMR and computational methodology in order to directly probe the orbitals involved in multiple boron–boron bonds experimentally *via* analysis of ^11^B–^11^B spin–spin (*J*) coupling constants. Computationally, the mechanism responsible for the boron–boron spin–spin coupling in these species is found to be analogous to that for the case of multiply-bonded carbon atoms. The trend in reduced *J* coupling constants for diborenes and a diboryne, measured experimentally, is in agreement with that known for alkenes and alkynes. This experimental probe of the electronic structure of the boron–boron multiple bond provides strong evidence supporting the originally proposed nature of the bonds in the diboryne and diborenes, and demonstrates that the orbitals involved in boron–boron bonding are equivalent to those well known to construct the multiple bonds between other second-row elements such as carbon and nitrogen.

## Abstract 2

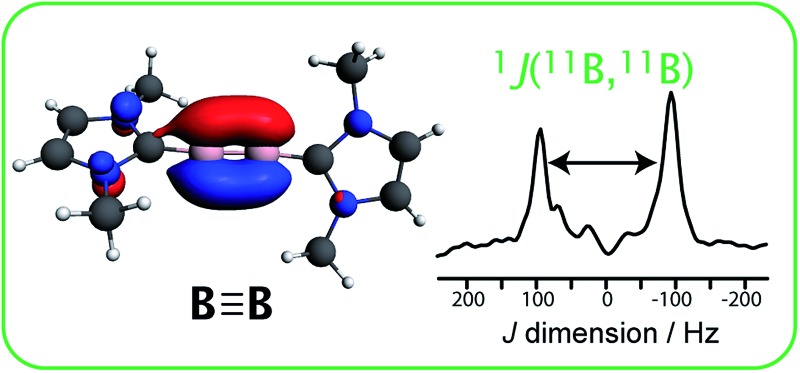
Boron–boron *J* coupling constants provide new insight into the nature of the boron–boron triple bond.

Although homonuclear multiple bonding is fairly common in the case of carbon and nitrogen, only recently has multiple bonding been demonstrated between two boron atoms in a neutral compound. With only three valence electrons it is impossible to construct a neutral diborene (compound with a boron–boron double bond) or diboryne (compound with a boron–boron triple bond) out of conventional electron-sharing bonds while simultaneously satisfying the octet rule. Power circumvented this issue electrochemically in the syntheses of dianionic diborenes ([R_2_B=BR_2_]^2–^) through the reduction of organodiboranes.^1^ More recently, a number of examples of neutral diborenes have emerged in the literature,^2,3^ as well as the first example of a stable diboryne,^4^ in which boron–boron multiple bonds are stabilised by neutral Lewis bases, such as carbenes and phosphines, which provide the extra electron missing from the normal valence of boron. The respective planar and linear geometries of diborenes and diborynes, which are well reproduced by their DFT calculated electronic structures,^5,6^ differ significantly from the geometries of their heavier group III analogues, digallyne^7^ and dialuminyne.^8^ This is much the same as the geometrical differences found between planar alkenes and linear alkynes and their heavier analogues in group IV,^9^ suggesting that boron–boron multiple bonding is closely related to multiple bonding in carbon and nitrogen. Indeed, quantum chemical calculations describe the orbitals of NHC-stabilized boron–boron multiple bonds as closely resembling the ubiquitous combination of σ- and π-bonds commonly understood to constitute the bonding in unsaturated organics and multiply bonded nitrogen species.^5,10^ However, despite the support from theory and the overall conceptual ease with which a triple bond between two boron atoms slots into long-known trends in the main group, the assignment of the triple bond in the diboryne has recently been disputed by Köppe and Schnöckel, who contend that the force constant of the boron–boron bond is lower than expected for a triple bond.^11^ They instead suggest, on the basis of calculated vibrational data, that the bond order is only slightly larger than 1.5. Subsequent to the report by Köppe and Schnöckel, experimental vibrational analysis by Raman spectroscopy and deconvolution of the relevant modes into relaxed force constants indicated that the vibrational frequencies and force constants of the B≡B bond are in agreement with the trends established by C≡C and N≡N bonds.^12^

As opposed to the use of force constants, which may be used to comment on the strength of a bond, the actual electronic structure of bonding orbitals which dictate the bond order can be experimentally probed with the use of indirect nuclear spin–spin (or *J*) coupling, which is most often measured by nuclear magnetic resonance (NMR) spectroscopy.^13^ The measurement of *J* coupling between quadrupolar nuclei such as ^11^B is unfortunately extremely challenging since the relevant spectral fine structure is typically obscured by the line broadening caused by quadrupolar relaxation in solution or by anisotropic second-order quadrupolar broadening in the solid state. However, new experimental methodology has recently been developed^14^ that enables the facile measurement of homonuclear *J* coupling involving half-integer quadrupolar nuclei and has been applied to the characterisation of the electronic structure of boron–boron single bonds as well as metal–metal bonds.^15^ Notably, it was demonstrated that information regarding the strength of the σ_BB_ bond, as well as its s-character, could be obtained by measuring the *J* coupling between the boron atoms. In those cases, the *J* coupling was affected by the electron-withdrawing capacity of the ligands, which acts to increase the s-character of the boron–boron σ-bond *via* an effect known as Bent's rule.^16^

The impact of multiple bonding on the *J* coupling between second-row atoms (such as carbon and nitrogen) is very well understood. The often dominant Fermi-contact (FC) *J* coupling mechanism is only non-zero for orbitals having significant s-character at both nuclear sites.^17^ As bond order increases, so does the s-character of the σ-bond since the hybridization state of the atom progresses from sp^3^ to sp^2^ and sp with the inclusion of additional π-bonding orbitals.^18^ The reduction in bond length associated with a stronger multiple bond also increases the orbital overlap, which serves to further increase the observed *J* coupling constant. Information about the electronic structures of boron–boron multiple bonds, which are predicted to behave similarly to bonds involving carbon, is thereby experimentally accessible through analysis of *J*(^11^B,^11^B) coupling.

We have measured the *J*(^11^B,^11^B) coupling in the diborene and diboryne compounds depicted in Scheme 1. Compound **1** is a diboryne featuring a boron–boron triple bond stabilised by two N-heterocyclic carbenes (NHC).^4^ Compound **2** is similar to **1**,^19^ with the exception that the significantly higher π-acidity of the stabilising cyclic (alkyl)(amino)carbene (CAAC)^20^ ligand leads to a species with the electronic structure and geometry of an electron deficient cumulene. Lastly, compounds **3** and **4** are NHC-stabilised diborenes flanked by either 2,3,5,6-tetramethylphenyl (**3**)^21,22^ or 2-thienyl (**4**) substituents.^23,24^ Together with the previously-studied diboranes,^15^ this represents a complete series of boron compounds whose *J*(^11^B,^11^B) values can be measured and directly compared with *J*(^13^C,^13^C) data for organic alkanes, alkenes, cumulenes, and alkynes.^25^

**Scheme 1:**
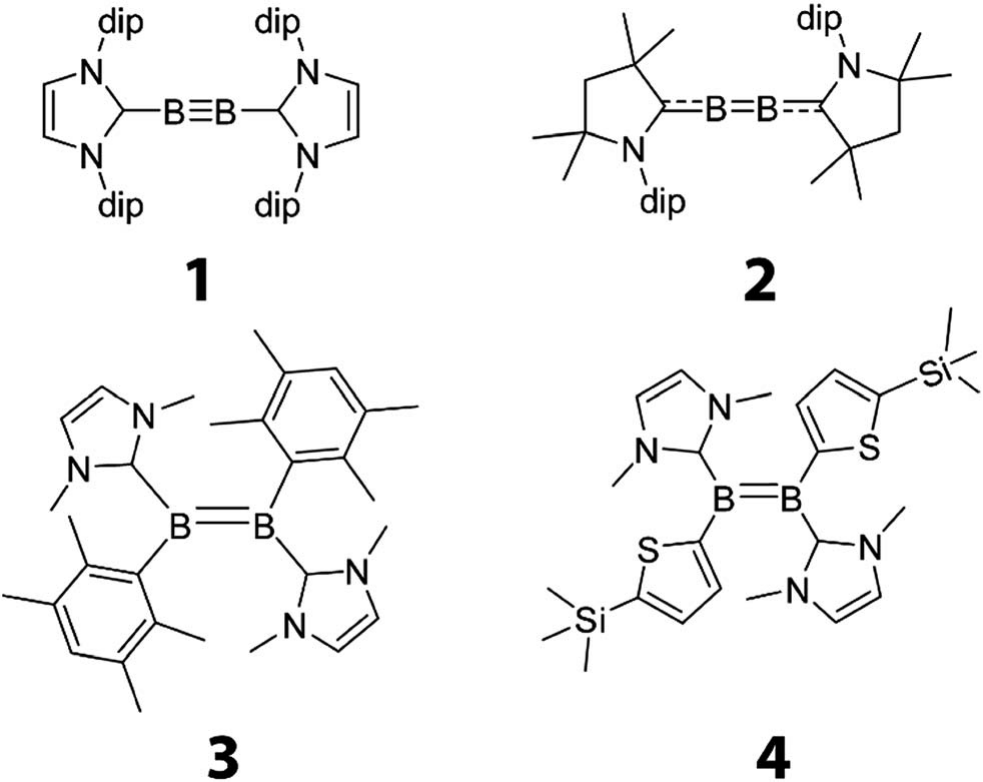
The boron–boron multiply bonded compounds.

Magic-angle spinning (MAS) double-quantum filtered (DQF) *J*-resolved NMR experiments^14^ were performed on samples **1** to **4** in order to determine their *J*(^11^B,^11^B) coupling constants; the spectra are shown in Fig. 1. In the case of **3**, double-quantum filtration led to a complete loss of signal, and thus a regular two-pulse *J*-resolved experiment was performed. Due to the quadrupolar nature of ^11^B, this leads to the appearance of an undesirable resonance at zero frequency.^26^ In all cases, however, a clear doublet can be observed whose splitting equals the *J* coupling constant, with the exception of **4**. As compound **4** has crystallographic inversion symmetry, the *J* splitting in a DQF-*J*-resolved experiment is amplified by a factor of 3 (Fig. 1, top) due to increased spin state mixing as previously described.^27^ The values for the *J* coupling constants are listed in Table 1.

**Fig. 1:**
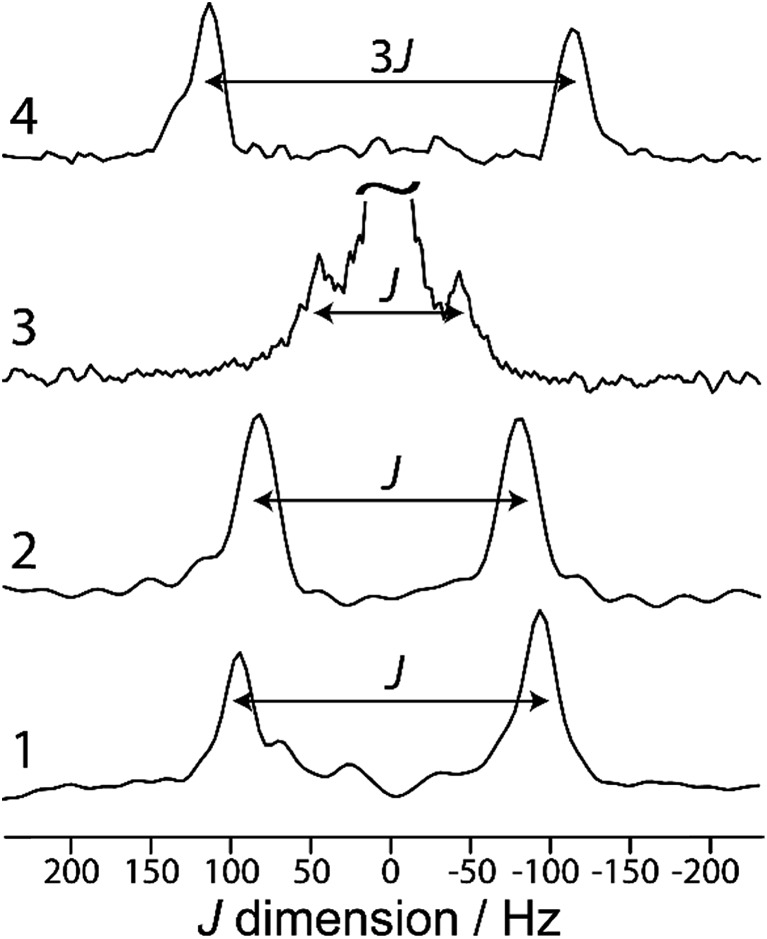
Indirect dimension of the 2D ^11^B DQF-*J*-resolved spectra acquired for compounds **1–4**, as indicated on the figure. The splitting of the resonances equals the *J* coupling constant, with the exception of **4**, for which the splitting equals 3*J* due to the magnetic equivalence of the ^11^B spins.

**Table 1:** *J*(^11^B,^11^B) coupling constants measured from experiment and predicted by DFT

Compound	exp. *J*(^11^B,^11^B)/Hz	PBE/TZP^28^ *J*(^11^B,^11^B)/Hz
**1**	187 ± 5	196.4
**2**	164 ± 5	167.8
**3**	85 ± 10	73.9
**4**	75 ± 3	65.7

In order to gain a greater insight into the origins of the *J* coupling in these compounds, and relate the results to the nature of the boron–boron bond, we have decomposed the DFT-calculated *J* coupling constants in terms of natural localised molecular orbital (NLMO) contributions.^29^ The calculated *J* coupling constants are listed in Table 1 and are in good agreement with experiment. NLMOs are highly localised molecular orbitals that are arranged to represent Lewis-type structures.^30^ There are NLMOs representing lone pairs, core functions, as well as σ- and π-bonds. The σ_BB_, π_BB_, and core s_B_ NLMOs for all four compounds are shown in Fig. 2. In all cases, the π_BB_ NLMOs have some degree of delocalisation with the carbene ligand, indicative of a degree of π-back-donation from the B_2_ moiety. As expected, this delocalisation is larger for **2** than **1** due to the CAAC's greater π acidity and the cumulene-type electronic structure for this species.^20^ The calculated degree of delocalization of the π-orbitals between B and C in **1** has been found to vary, often significantly, with the method and level of computation,^4,5^ though the physical significance of this delocalisation is perhaps minimal. As is the case here, in computations using small NHC-ligands, the minimized geometries show orthogonal alignment of the NHC ligands, with the planes of the NHC rings at 90° angles to one another. However, the crystallographically determined structure of **1** shows an interplanar angle of ∼56°.^4^ A very similar diboryne has recently been reported wherein the isopropyl arms on the phenyl substituent of the bulky NHC are swapped for slightly smaller ethyl groups. This slight change in the bulk resulted in a large change in the interplanar angle (∼85°), with minimal effect on the B≡B and B–C bonds, which were identical within crystallographic error.^12^ A computational study on the effect of changes in the interplanar angle on the length of the B≡B and B–C bonds, as well as on the electronic energy of the compound, showed very little overall influence.^12^ The insensitivity of the C–B–B–C core in **1** to changes in NHC orientation, which should be large if B → C backdonation were energetically important, speak to the alkyne-like orbital construction of **1**. The CAAC ligands in **2** are nearly orthogonal, as would be expected for a cumulene deficient by two electrons.^19^

**Fig. 2:**
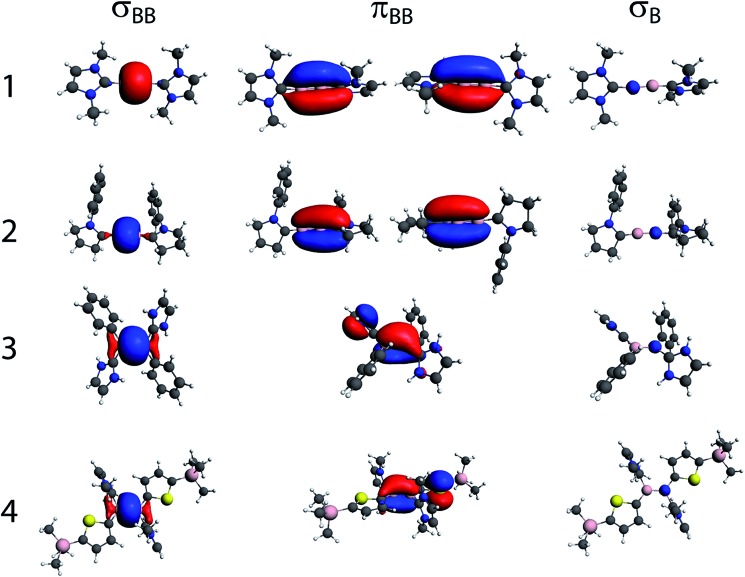
The bonding (σ and π) and core NLMOs for the multiply-bonded boron compounds from Scheme 1.

As is the case for ^13^C–^13^C *J* coupling, the NLMO analysis shows that the π_BB_ orbitals do not contribute to *J* coupling and that most of the *J* coupling originates from the σ_BB_ bonding orbital and the core s functions on the boron nuclei (the decomposition of the *J* coupling in terms of NLMO is given in Table S1†). As mentioned earlier, this is due to the fact that p-type orbitals cannot contribute to the *J* coupling through the FC mechanism. The *J* coupling is much larger for compounds **1** and **2** (187 and 164 Hz, respectively), which feature sp-hybridized boron atoms, than for compounds **3** and **4**, which are sp^2^-hybridized (85 and 75 Hz, respectively). As is intuitive (and as has been substantiated for diboranes^14^), the *J* coupling is stronger in the case of the triply-bonded compound due to its shorter internuclear distance than in compound **2** (1.449(3) *vs.* 1.489(2) Å), in which the spread of the π-system results in lower overall bond order between the boron atoms.^19^ The effects from Bent's rule when comparing these two compounds are also negligible since they have nearly identical boron–boron bonding s-character, as shown in Table S1.†

Perhaps surprisingly, the *J* coupling is weaker in the case of these diborene systems than in the singly-bonded systems that have been previously studied by us (*J*(^11^B,^11^B) ranging from 98 to 130 Hz).^15^ However, this is easily explained by the fact that the boron nuclei of the diborene are bound to carbon, as opposed to oxygen in the case of the diborane compounds. As a result of the greater electron withdrawing capacity of oxygen, the s-character of the diborane σ_BB_ orbital is significantly increased in accordance with Bent's rule. For comparison, the *J*(^11^B,^11^B) value for tetramethyldiborane was calculated as 55 Hz, somewhat smaller than the values for the doubly-bonded compounds studied here.^15^

Although these data convincingly show a significant increase in the bond order of the boron–boron bond in compound **1** as compared to the diboracumulene (**2**) and the characterised diborenes (**3** and **4**), it is additionally possible to directly compare the carbon–carbon and boron–boron coupling with the use of reduced *J* coupling constants (*K*). *K* is defined as 4π^2^ *J*/*γ*_1_ *γ*_2_ *h* and is an isotope-independent *J* coupling constant. In similar bonding environments, the *K* values are known to be approximately proportional to the product of the atomic numbers of the two coupled nuclei and thus *K*(B,B) values are expected to be on the order of 25/36(0.69) times weaker than the corresponding *K*(C,C) values.^31^ Given that the *J*(^13^C,^13^C) value in acetylene is 171.5 Hz (*K* = 225.89 × 10^19^ N A^–2^ m^–3^), the expected *J*(^11^B,^11^B) coupling constant in a diboryne having an equivalent bonding structure would be approximately 193.9 Hz (*K* = (25/36) × 225.89 × 10^19^ N A^–2^ m^–3^); this is in excellent concurrence with the experimental value of 187 ± 5 Hz.

Fig. 3 shows a plot of the experimental *K*(B,B) values for compounds **1** to **4**, as well as the calculated value for B_2_(Me)_4_,^15^ as a function of the experimental *K*(C,C) values for ethane, ethene, ethyne,^25^ and diacetylene.^32^ Diacetylene was chosen as a model due to its electronic similarity to butatriene whose *J*(^13^C,^13^C) coupling constants have not been measured. The calculated value of the *J* coupling in butatriene is also similar to that in diacetylene.^33^ As can be seen in Fig. 3, the *K*(B,B) and *K*(C,C) values are strongly correlated and the experimental slope of the regression fit is 0.60; very close to the expected slope of approximately 0.69. The *K*(B,B) values are systematically larger by 13.9 N A^–2^ m^–3^ because the boron atoms are bonded to carbon as opposed to hydrogen, which results in slightly larger *J* coupling by Bent's rule.^15^

**Fig. 3:**
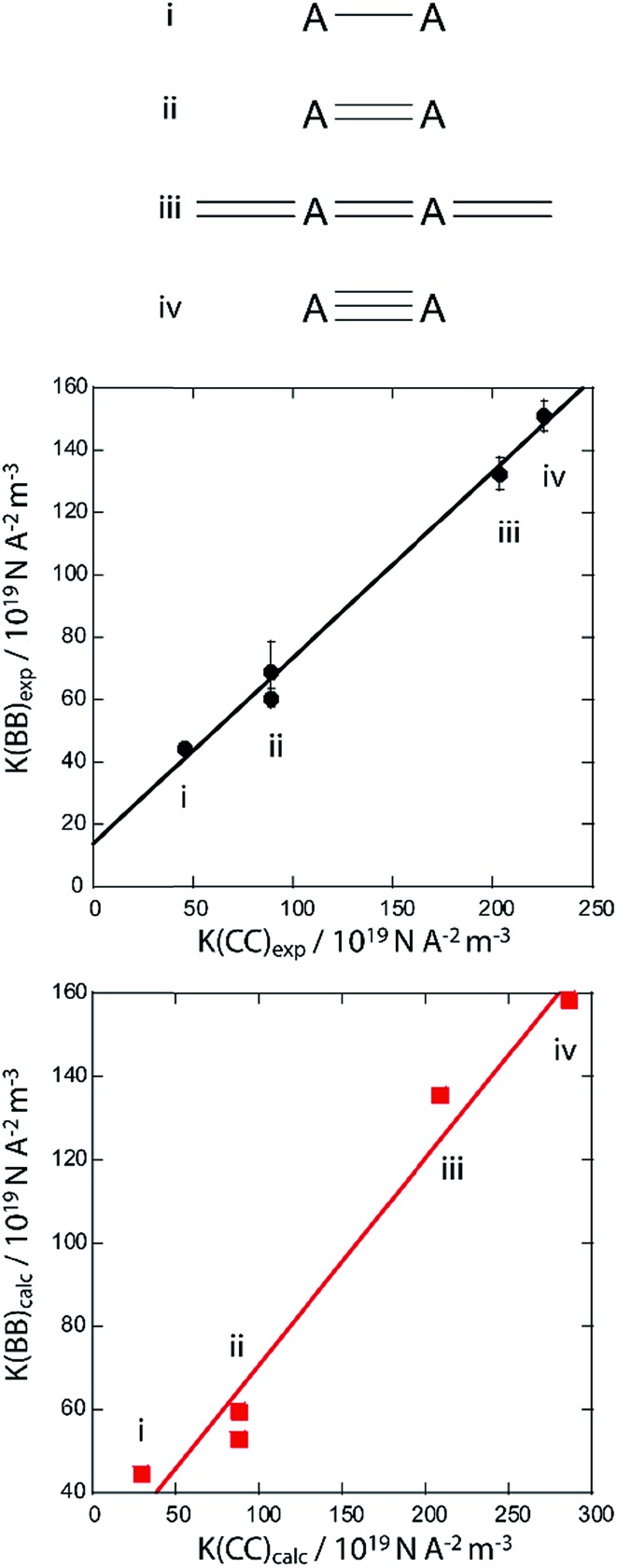
Plot showing the correlation between the reduced *J* coupling constants between carbon atoms in ethane (i), ethylene (ii), diacetylene (iii), and acetylene (iv) and those between boron atoms in B_2_Me_4_ (i, calculated), **3** and **4** (ii), **2** (iii), and **1** (iv). Data obtained from experiment are shown in black (middle) and PBE/TZP calculated values are in red (bottom). The experimental data are fit by the expression: *K*(B,B) = 0.595*K*(C,C) + 13.9 × 10^19^ N A^–2^ m^–3^ (*R* = 0.99613) whereas the calculated data are fit by the expression: *K*(B,B) = 0.498*K*(C,C) + 20.9 × 10^19^ N A^–2^ m^–3^ (*R* = 0.98307). Structures showing the typical bonding arrangement in the compounds are shown on the top where A = carbon or boron.

In conclusion, we were able to show experimentally that multiple boron–boron bonding behaves analogously to that of the other elements of its row in the periodic table (*i.e.*, C, N, and O), as opposed to the heavier icosagens (Ga and Al). The increase in bond order from 2 to 3 leads to an increase in the s-character of the bond and in the observed *J*(^11^B,^11^B) coupling constants. Both the hybridization of the atoms and the variation of the s-character of the bond by the ligands, *via* Bent's rule, determine the magnitude of the coupling constant. Quantitative agreement is found when directly comparing the reduced *J* coupling constants in analogous diboron and carbon–carbon bonded organic species, once the differences in atomic numbers are considered, indicating that the B–B bonding orbitals mirror those that are well known for carbon multiple-bonding. This result, as well as the significant increase *J* coupling when comparing **1** and **2**, supports the previous characterisation of compound **1** as a diboryne.
