# Supplementary material for: Spying on the boron–boron triple bond using spin–spin coupling measured from 11B solid-state NMR spectroscopy
Source: Chem Sci. 2015 Apr 1;6(6):3378–82. doi: 10.1039/c5sc00644a (PMC5657093; doi:10.1039/c5sc00644a)
Supplement: Supplementary file 1 [file SC-006-C5SC00644A-s001.pdf]

Electronic Supplementary Material (ESI) for Chemical Science.
This journal is © The Royal Society of Chemistry 2015

ELECTRONIC SUPPLEMENTARY INFORMATION

Spying on the Boron-Boron Triple Bond Using Spin-Spin Coupling Measured from ^{11}B Solid-State NMR Spectroscopy

Frédéric A. Perras,[1,§] William C. Ewing,[2] Theresa Dellermann,[2] Julian Böhnke,[2] Stefan Ullrich,[2] Thomas Schäfer,[2] Holger Braunschweig,[2,*] and David L. Bryce[1,*]

[1] Department of Chemistry and CCRI, University of Ottawa, 10 Marie Curie Pvt. D'Iorio Hall, Ottawa, Ontario K1N6N5, Canada.

[2] Institut für Anorganische Chemie, Julius-Maximilians-Universität Würzburg, Am Hubland, 97074, Germany.

[§]Present Address: Ames Laboratory, U.S. Department of Energy, Ames, Iowa 50011, United States

*Authors to whom correspondence may be addressed:

dbryce@uottawa.ca

h.braunschweig@uni-wuerzburg.de

Experimental

Synthesis

Compounds **1**,[1] **2**,[2] **3**,[3] and **4**[4] were prepared as previously described.

Solid-State NMR Spectroscopy

For all NMR experiments, the samples were packed into 4 mm o.d. MAS rotors under an inert atmosphere immediately prior to running the experiments. The experiments were performed using a Bruker AVANCE 500 NMR spectrometer using a Bruker triple-resonance 4 mm MAS probe. The ^{11}B *J*-resolved and DQF-*J*-resolved spectra were obtained using the previously published pulse sequences[5,6] and used 15 μs central-transition selective 90° pulses and a DQF time of 4 to 6 ms. A 2 s recycle delay was used in all cases and the samples were spun at a 10 kHz MAS frequency. Between 25 and 42 t_1 increments of 500 μs were performed for the 2D acquisition; between 128 and 2048 scans were acquired in each increment. The final spectra were obtained by magnitude processing.

---

1 H. Braunschweig, R. D. Dewhurst, K. Hammond, J. Mies, K. Radacki, and A. Vargas, *Science*, 2012, **336**, 1420.

2 J. Böhnke, H. Braunschweig, W. C. Ewing, C. Hörl, T. Kramer, I. Krummenacher, J. Mies, and A. Vargas, *Angew. Chem. Int. Ed.* 2014, **53**, 9082.

3 P. Bissinger, H. Braunschweig, A. Damme, T. Kupfer and A. Vargas, *Angew. Chem. Int. Ed.* 2012, **51**, 9931.

4 H. Braunschweig, R. D. Dewhurst, C. Hörl,. A. K. Phukan, F. Pinzner and S. Ullrich, *Angew. Chem. Int. Ed.* 2014, **53**, 3241.

5 F. A. Perras and D. L. Bryce, *J. Am. Chem. Soc.* 2013, **135**, 12596.

6 F. A. Perras and D. L. Bryce, *J. Phys. Chem. Lett.* 2014, **5**, 4049.

DFT Calculations

All DFT calculations were performed using the Amsterdam Density Functional program (ADF, ver. 2009).[7] DFT calculations were performed using the generalised gradient approximation (GGA) DFT functional of Perdew, Burke, and Ernzerhof (PBE)[8] using triple-zeta polarised Slater-type basis sets (TZP). The NBO/NLMO analysis was performed using the NBO 5.0 program,[9] which is included with ADF. The results of the NLMO decomposition of the *J* couplings are given in Table S1.

Table S1. Results from the NLMO analysis of the $J(^{11}B, ^{11}B)$ coupling constants

Compound	σ_{BB} contribution to J / %	s_B contribution to J / %	σ_{BB} s-character / %	σ_{BB} NBO energy / a.u.
**1**	33.1	64.4	52.5	-0.399
**2**	36.3	74.0	50.7	-0.410
**3**	34.6	69.7	32.5	-0.309
**4**	33.9	81.4	31.5	-0.314

[7] G. te Velde, F. M. Bickelhaupt, E. J. Baerends, C. Fonseca Guerra, S. J. A. van Gisbergen, J. G. Snijders and T. Ziegler, *J. Comput. Chem.*, 2001, **22**, 931.
[8] J. P. Perdew, K. Burke, M. Ernzerhof, *Phys. Rev. Lett.* 1996, **77**, 3865.
[9] E. D. Glendening, J. K. Badenhoop, A. E. Reed, J. E. Carpenter, J. A. Bohmann, C. M. Morales and F. Weinhold, NBO 5.0., Theoretical Chemistry Institute, University of Wisconsin, Madison, WI, 2001.
